# Improving Hemocompatibility of Polysulfone Membrane by UV-Assisted Grafting of Sulfonated Chitosan

**DOI:** 10.3390/polym16111555

**Published:** 2024-05-31

**Authors:** Sheng Yan, Yunren Qiu

**Affiliations:** College of Chemistry and Chemical Engineering, Central South University, Changsha 410083, China; ysheng326@163.com

**Keywords:** polysulfone, chitosan, graft, hydrophilicity, hemocompatibility

## Abstract

The most prevalent type of hemodialysis membrane is polysulfone (PSf). However, due to inadequate biocompatibility, it significantly compromises the safety of dialysis for patients. In this study, we modify the surface of the PSf membrane with 2,4-dihydroxybenzophenone (DBPh) groups to serve as anchoring sites during UV irradiation. Subsequently, a tailored sulfonated dihydroxy propyl chitosan (SDHPCS) is grafted onto the modified PSf membrane to compensate for the deficiencies in hydrophilic additives. The modified PSf membrane exhibits outstanding hydrophilicity and stability, as demonstrated by its characterization and evaluation. This paper focuses on investigating the interaction between platelet membrane formation, protein adsorption, and anticoagulant activity. The results show that the modified PSf membrane exhibits remarkable enhancement in surface hydrophilicity, leading to a significant reduction in protein and platelet adsorption as well as adhesion.

## 1. Introduction

The polysulfone (PSf) and PSf-based membranes exhibit exceptional chemical, thermal, and hydrolytic stability, outstanding strength, a high glass transition temperature, as well as favorable membrane-forming and mechanical properties [1,2]. They are extensively utilized in the field of biomedicine, particularly for implantable devices [3], devices that come into contact with blood [4,5], and biosensors [6]. The synthesis and applications of PSf and PSf-based membranes have made significant advancements; however, the hydrophobicity of polysulfone limits its application [4,7]. In particular, the PSf membrane is employed in hemodialysis, when it comes into contact with blood, which can result in rapid protein adsorption onto the membrane surface, and this adsorbed protein layer may subsequently lead to platelet adhesion, aggregation coagulation, and other further undesirable outcomes [8,9]. The incorporation of functional groups into PSf not only overcomes these limitations but also expands the range of potential applications for these high-performance materials by imparting specific properties, thereby providing a broader scope for their application [10,11]. Despite the extensive literature on material surface modification strategies, challenges remain in achieving the efficient installation of functional groups and avoiding undesirable side effects resulting from harsh reaction conditions [12,13,14]. The UV-induced surface modification of PSF membranes is characterized by a simple operation and controllable reaction. The fabrication of the PSf membrane is achieved through the feasible UV-induced photo-grafting polymerization of diallyl dimethyl ammonium chloride (DADMAC). The obtained graft copolymers exhibit outstanding antifouling properties and favorable hydrophilicity [15]. The obtained membrane exhibits excellent hydrophilicity and high flux [16]. The PSF hollow fiber membrane was subjected to graft polymerization with 2-Hydroxyethylacrylate initiated by ultraviolet radiation, resulting in a significant enhancement of the hydrophilicity and fouling resistance of the modified membrane [17].

Although there exists an extensive body of work in the literature on strategies for UV-initiated material surface modification, the construction of a dense polymer brush coating on nonpolar polymeric surfaces remains challenging due to the absence of functional surface groups. The primary challenge in the construction of polymer brushes lies in achieving a high grafting density, which induces lateral steric repulsion to extend folded polymer chains into a brush conformation. To address this issue, 2,4-dihydroxybenzophenone (DBPh) is utilized as a surface treatment reagent due to its aliphatic C-H insertion chemistry for tethering polymer chains onto the surfaces of polysulfone membranes [18]. The DBPh groups are covalently linked with polysulfone membrane via photoinduced grafting and cross-linking. In this way, the hydroxyl group of DBPh is introduced onto the surface of the PSf membrane, which is conducive to the further modification of the PSf membrane.

Therefore, for the first time, the keto group of 2,4-dihydroxybenzophenone was inserted into aliphatic C-H on the surface of the polysulfone membrane by a UV-controlled grafting technique to form a covalent chain. Under ultraviolet irradiation (254 nm/365 nm), the keto group undergoes an π-π * transition and is excited to a single linear state S. And then, it rapidly transitions to a triplet state to seize hydrogen atoms from the surface of the adjacent polysulfone membrane and covalent links to the surface of the membrane, thus anchoring the active functional groups (hydroxyl groups) that are easy to graft modifications onto the surface of the membrane.

The biocompatible polymer chitosan possesses remarkable properties that make it highly suitable for a wide range of biomedical applications [19,20]. The increasing utilization of chitosan in the medical field necessitates a comprehensive description of its interactions with blood [21]. The majority of studies have indicated that the interactions between the free amino groups of chitosan and plasma proteins or blood cells may potentially trigger thrombosis or hemolytic responses [22]. The findings of some studies indicate that chitosan exhibits a high propensity for thrombosis [23,24]. The oxygen, 5-carbon sugar and the surrounding amino and hydroxyl functional groups of the main chain of chi-tosan can be easily modified chemically to improve the hydrophilicity and anticoagulant properties of chitosan [25]. The sulfonic acid group, ammonium sulfate group, and carboxylic acid group are modified to the structure of chitosan to control the charge and molecular size of modified chitosan, which can not only avoid the side effects of chitosan, but also obtain a heparin-like state of hydrophilicity and anticoagulant activity. The sulfonated dihydroxy propyl chitosan (SDHPCS), a great potential heparin-like substance, is obtained via hydroxylation and sulfonation reaction, which has prominent hydrophilicity and anticoagulant properties that can be attributed to the heparin-like structure.

After contact between blood and the dialysis membrane materials, the blood stabilization mechanism is destroyed, leading to a cascade reaction of a platelet-centered coagulation system, thrombosis, and embolism, which seriously endangers patients’ lives. Therefore, anticoagulation therapy is a necessary means of blood purification treatment to reduce thrombosis. Clinical practice is to use drugs for systemic anticoagulation therapy. For high-risk bleeding patients, the risk of bleeding and thrombosis during dialysis is difficult to balance, so dialysis membrane materials can only be modified to ensure that the risk of systemic bleeding of patients is not increased. The occurrence of thrombosis in the blood contact of dialysis membrane needs to be reduced. Therefore, the blood compatibility requirements of polymer biomaterials are increasingly high, in order to achieve the contact between blood and membrane materials, not produce the coagulation phenomenon, and activate the immune system’s stability. It is necessary to understand the interaction between polymer materials and blood at the molecular level and the cellular level, through the in-depth study of the relationship between the structure of the material and anticoagulation, and from the perspective of molecular design, produce synthetic high molecular blood compatibility materials with better blood compatibility. From the perspective of chemical structure, there are no hydrophilic groups and anticoagulant molecules in the structure of the polysulfone membrane, which causes the risk of protein adsorption, platelet adhesion, thrombosis, and allergic reaction after contact with blood, limiting the development and application of polysulfone membranes in the field of hemodialysis. The active site was anchored onto the surface of the polysulfone membrane by a UV-controlled grafting technique. The problem that it is difficult to construct a high-density chitosan thin layer on the surface of polysulfone is overcome. The realization of an ultra-thin, smooth, highly stable single-layer brush coating opens up a new way of modifying polysulfone film and the in situ fixation of a variety of selective molecules.

**Scheme 1:**
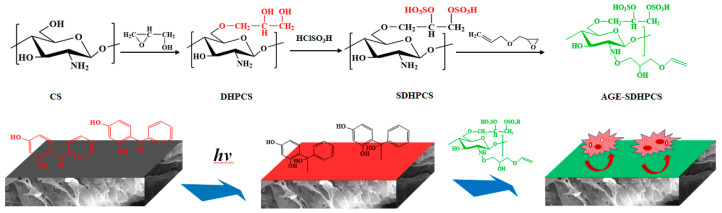
The synthesis procedure of graft 2,4-dihydroxybenzophenone and sulfonated dihydroxy propyl chitosan onto the PSf membrane surface.

In this study, dihydroxy propyl chitosan was first prepared from chitosan, and then sulfonated under appropriate conditions to obtain a good anticoagulant SDHPCS. In order to reduce the spatial repulsion between SDHPCS and PSf membrane and improve the graft rate of SDHPCS on the surface of the PSf membrane, the allyl glycidyl ether (AGE) was introduced into SDHPCS as the lateral extension chain. And then, the hydroxylated PSF membrane (PSf-OH) was obtained so that the DBPh groups were grafted to the surface of the PSf membrane by photoinduced grafting. The PSf-SDHPCS was obtained where the SDHPCS was covalently linked with the PSf-OH membrane via surface chemical reactions. The detailed synthetic routine is shown in Scheme 1.

## 2. Objects and Methods

### 2.1. Materials

Polysulfone (PSf, average Mn: 22,000), allyl glycidyl ether (AGE), (±)glycidol, and formamide were purchased from Rhawn. The degree of deacetylation of chitosan (CS) was about 95% and chlorosulfonic acid and 2,4-dihydroxybenzophenone (DBPh) were provided by Aladdin Industrial Corporation. Acetone, isopropanol, absolute ethanol, and hydrochloric acid were of analytical grade were purchased from Sinopharm Group Chemical reagent Co., LTD, Shanghai, China.

### 2.2. Preparation of Sulfonated Dihydroxy Propyl Chitosan (SDHPCS)

The SDHPCS was synthesized by the following steps: First, dihydroxy propyl chitosan (DHPCS) was synthesized. Simply, 4 g dried alkaline chitosan was put into a 150 mL three-neck bottle, 40 mL isopropyl alcohol was slowly added, mixed and stirred for 40 min, then, an appropriate amount of epoxy propyl alcohol was added, and reacted under the protection of nitrogen at a certain temperature. After the reaction, add the right amount of high purity water to stir for dissolution, after cooling, centrifuge at 4000 r·min^−1^ for 10 min, then adjust the pH value with dilute hydrochloric acid to neutral, and then add acetone to precipitate a large number of white precipitates. After the reaction was over, 100 mL ultra-pure water was added to the flask for dilution after cooling. If there were unreacted solid substances, the unreacted ones were fixed and filtered. Then, the reaction solution was transferred to the dialysis bag treated with the deoxygenation layer for static dialysis for 24 h. Then, NaOH solution was added to the dialysate to adjust the pH to more than 12, and the deamination was carried out for 30 min, and hydrochloric acid solution was added to adjust the pH to 3~5. The dialysate was then transferred to the newly deoxidized dialysis bag for static dialysis for more than 48 h. Then, the dialysate was fine vacuum-dried to obtain SDHPCS.

### 2.3. Sulfonated Dihydroxy Propyl Chitosan-Grafted Allyl Glycidyl Ether (AGE-SDHPCS)

A total of 2 g of SDHPCS was dissolved in 20 mL ultra-pure water and 20 mL allyl glycidyl ether solution was added; the reaction was carried out at 60 °C for 2 h. After the reaction, add 20 mL of water for extraction, remove the lower layer liquid, then vacuum dry to obtain a brown–yellow solid powder.

### 2.4. PSf Membrane Grafted with 2,4-Dihydroxybenzophenone (PSf-OH)

The PSf membrane was soaked in a certain concentration of 2,4-dihydroxybenzophenone (DBPh) solution at room temperature for a certain period of time. Then, under N_2_ protection, the DBPH-coated polysulfone membrane was irradiated under a light-emitting diode (LED) UV lamp (365 nm, 30 mW·cm^−2^) for a period of time, and the PSf membrane was thoroughly rinsed with methanol and ultra-pure water. It was dried with N_2_ flow.

### 2.5. Synthesis of PSf-OH Membrane Grafted with SDHPCS (PSf-SDHPCS)

The activated materials were immersed in an acetic acid solution with 0.5 mg·mL^−1^ sulfonated dihydroxy propyl chitosan (AGE-SDHPCS), 0.2 g ammonium persulfate and PSf-OH membrane were added under nitrogen protection at 25 °C for 2 h, 3 h, 4 h, 5 h, and 6 h. The materials were washed with phosphate-buffered saline and deionized water, filtered and vacuum dried under room temperature, and the SDHPCS-modified PSf membranes were obtained.

### 2.6. Characterization

The surface chemical structures of the modified membranes were characterized by Fourier transform infrared spectroscopy (FTIR). The surface element composition was detected by X-ray photoelectron spectroscopy (XPS) using Al Kα excitation radiation (1486.6 eV). The cross section morphology of the membrane was determined by scanning electron microscopy (SEM) at 5 kV.

### 2.7. Blood Compatibility

#### 2.7.1. Bovine Serum Albumin (BSA) Adhesion

The modified polysulfone membrane was cut into a membrane of 1 cm × 1 cm, and then soaked in phosphate-buffered saline with pH = 7.4 for 24 h. After soaking, the membrane was removed and then soaked in a bovine serum albumin (BSA) solution at a temperature of 37 °C and a concentration of 1 mg·mL^−1^ for 2 h. The membranes were washed with a PBS and gently stirred in a 2 wt.% sodium dodecyl sulfate (SDS) solution at 37 °C to remove adsorbed proteins. BSA content in the sodium dodecyl sulfate (SDS) washing solution was measured by UV spectrophotometer at 280 nm.

#### 2.7.2. Hemolysis Test

First, the sample diaphragm (1 cm × 1 cm) was placed in the centrifuge tube, and then 2 mL of normal saline was introduced into the centrifuge tube, the normal saline was preheated at 37 °C for 1 h, and then gently poured out. Next, 10 mL of the blood sample was diluted into 60 mL of normal saline, and 3 mL of this dilute solution was introduced into each centrifuge tube. In addition, 2 mL of blood samples were added to 12 mL of pure water and saline to serve as positive and negative controls, respectively. Finally, all samples were kept in a water bath at 37 °C for 3 h, after which all centrifuges were transferred to the centrifuge and centrifuged at 500× *g* for 10 min. The supernatant after centrifugation was placed in an ultraviolet spectrophotometer and the absorbance was measured at 545 nm. Hemolysis rate (HR) was calculated as follows:

(1) $$HR=\frac{D_{t}-D_{nc}}{D_{pc}-D_{nc}}\times 100\%$$

where D_t_ is the absorbance of test sample; D_nc_ is the absorbance of the negative control; and D_pc_ is the absorbance of the positive control.

#### 2.7.3. Platelet Adhesion

The prepared PSf, PSf-OH, and PSf-SDHPCS membranes (1 cm × 1 cm) were immersed in normal saline at 37 °C for 1 h. The membranes were then placed into 5 mL test tubes, which were placed into a water bath at 37 °C. The prepared 1 mL PRR was introduced and incubated for 2 h. The membrane was removed, rinsed with normal saline three times, and placed in 2.5 wt. % glutaraldehyde saline. The platelets were fixed at 4 °C for 24 h. The membrane was then flushed again with saline. The samples were then soaked in alcohol solutions for 15 min each, and then soaked in isoamyl acetate–alcohol solutions for 15 min each; after complete dehydration, the samples were freeze-dried. After the PSf, PSf-OH, and PSf-SDHPCS membranes with fixed platelets were sprayed with platinum, the number and morphology of platelets sticking to the membrane surface were observed by SEM.

#### 2.7.4. Plasma Recalcification Time (PRT)

Anemic pulp (PPP) was prepared by taking fresh healthy human whole blood and centrifuging it at 3000 rpm for 15 min. The sample PSf, PSf-OH, and PSf-SDHPCS membranes were cut into 1 cm × 1 cm sample membranes, which were placed in PBS solution and incubated at 37 °C for 1 h, and then the membranes were cleaned with PBS. Put 0.5 mL of PPP, then put the test tube into the water bath at 37 °C, then add 0.025 mol·L^−1^ CaCl_2_ solution heated at 37 °C in pre-heating and start timing. Immerse the stainless steel hook in the PPP solution and observe and record the time when the first insoluble fibrin appears. This is called the plasma recalcification time.

#### 2.7.5. APTT, PT and TT

The prepared PSf, PSf-OH. and PSf-SDHPCS membranes (1 cm × 1 cm) were immersed in normal saline at 37 °C for 1 h. After that, the membranes were placed into 5 mL test tubes, and the test tubes were placed into the water bath device at 37 °C. The prepared 1mL PPP was introduced and incubated for 1 h. Then, the APPT, PT, and TT of plasma were measured by automatic hemagglutination apparatus.

## 3. Results and Discussions

### 3.1. Surface Characterization

#### 3.1.1. Synthesis of Chitosan Derivates

**Figure 1:**
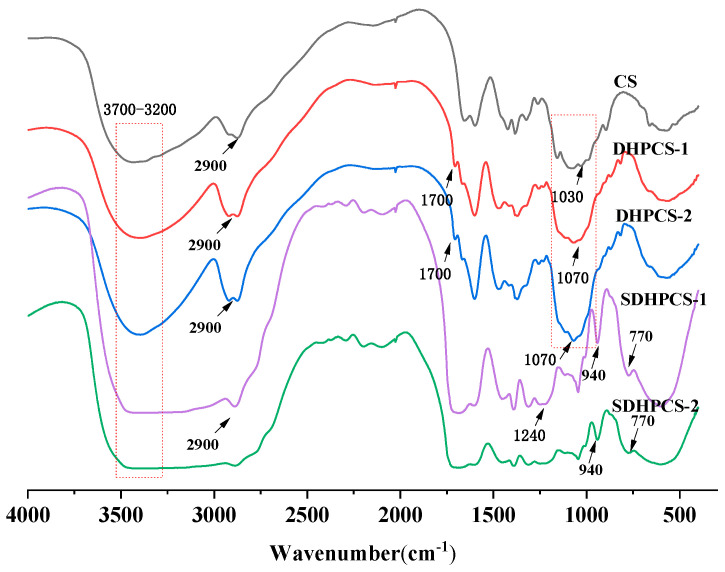
The FTIR spectra of CS, DHPCS, and SDHPCS.

FTIR tests were performed on the synthesized chitosan and its derivatives, and the test results are shown in Figure 1. The results showed that the alcohol hydroxyl stretching vibration peak appeared in the synthesized DHPCS at 1070 cm^−1^, and the peak intensity was larger than that of CS. Meanwhile, the -OH absorption peak of DHPCS was enhanced at 3200–3700cm^−1^. The enhanced absorption peaks at 2900 cm^−1^ ~ 2922 cm^−1^ indicate the presence of a large number of methylene hydroxyl groups in the molecular structure of DHPCS. The peak at 2900 cm^−1^ is the result of the C-H bond stretching vibration of the saturated hydrocarbon, indicating the presence of a large number of dihydroxy propyl groups in the synthesized derivatives. The peak value of C-O-C increased at 1018 cm^−1^ ~ 1180 cm^−1^ and the absorption of original C_6_-OH decreased significantly at 1030 cm^−1^, indicating that a large amount of dihydroxy propyl was grafted on C_6_-OH. The peak strength of the synthesized SDHPCS weakens or disappears at 1070 cm^−1^, and the characteristic peaks of the S=O bond stretching vibration appear at 1240 cm^−1^, 940 cm^−1^, and 770 cm^−1^. Meanwhile, the -OH absorption peaks of SDHPCS at 3200–3700 cm^−1^ also gradually weaken. In particular, the absorption peak of the dihydroxy propyl group at 2900 cm^−1^ ~ 2922 cm^−1^ almost disappeared. The -SO_3_H group successfully replaced the dihydroxy propyl group and grafted onto the molecular chain of chitosan.

#### 3.1.2. Water Contact Angle Analysis

**Figure 2:**
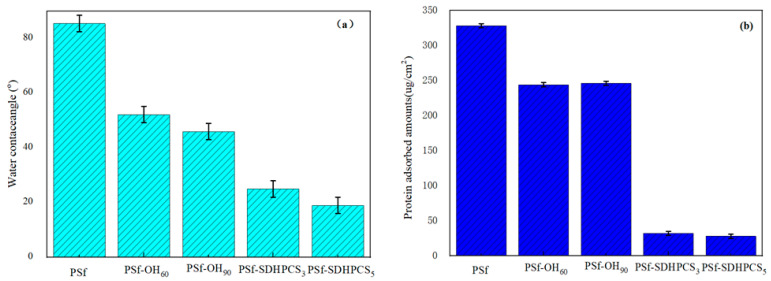
Static water contact angles for different membranes (**a**); the BSA adsorption on the surfaces of PSf, PSf-OH, and PSf-SDHPCS membranes (**b**). Data are expressed as the mean ± SD of six independent measurements.

In the modification evaluation of the hemodialysis membrane materials, assessing the hydrophilicity is one of the important methods of evaluating the blood compatibility after modification. Because of the highly hydrophilic membrane material, the adsorption of proteins on the membrane surface can be reduced. The static contact angle was used to evaluate the wettability of the membrane surface, and the water contact angle decreased with the increase in hydrophilicity, so as to evaluate the hydrophilicity of the surface of the modified membrane material. Because the surface contains polar or hydrophilic groups, it can form more hydrogen bonds with water, which is conducive to the transfer of water molecules. The static contact angle is a direct method of evaluating the wettability and decreases with the increasing hydrophilicity to indicate the hydrophilicity of the membrane surface [26]. Figure 2a shows that the water contact angle of the PSf, PSf-OH, and PSf-SDHPCS membrane surface was tested. The results showed that the contact angles of PSf and PSf-OH membranes were 85.4° and 63.8°, respectively, indicating that the unmodified PSf membrane is a non-hydrophilic membrane material. After it is directly in contact with blood, a large number of blood proteins will be adsorbed to the membrane surface and micro-thrombi will be formed. After grafting -OH, the water contact angle on the membrane surface decreased, indicating that DBPh also had a certain effect on hydrophilic modification. After the grafting of SDHPCS, the surface hydrophilicity of the PSf membrane was significantly reduced, and the contact angle of the PSf-SDHPCS membrane was 19.0°. It can be seen that the hydrophilicity of the SDHPCS-modified membrane is significantly improved.

#### 3.1.3. ATR-FTIR Analysis

**Figure 3:**
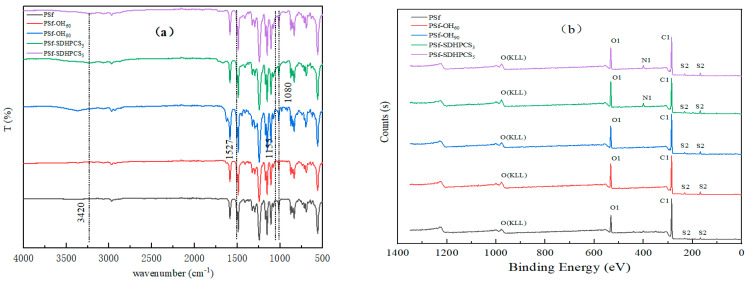

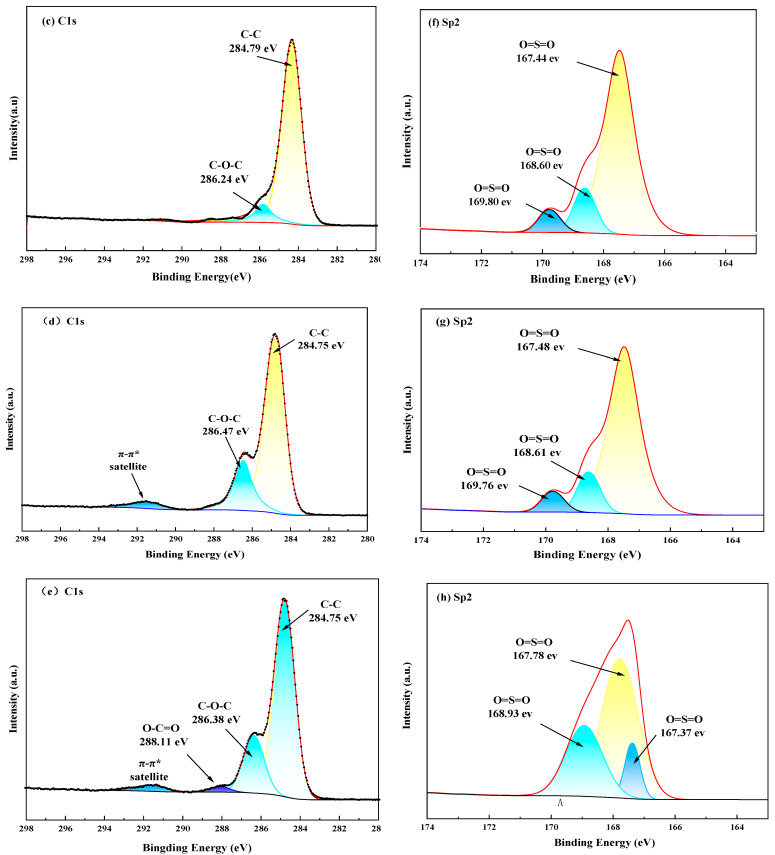
ATR-FTIR spectra (**a**) and XPS C1s and Sp2 high resolution spectra (**b**–**h**) of the PSf, PSf-OH, and PSf-SDHPCS membranes.

Figure 3a shows the ATR-FTIR spectra of PSf, PSf-OH, and PSf-SDHPCS membranes. The changes in functional groups on the surface of PSf, PSf -OH, and PSf-SDHPCS membranes were tracked by attenuated total reflection–Fourier transform infrared spectroscopy (ATR-FTIR) during the modification process. For PSf, the DBPh anchoring structure was grafted by UV. Compared with the infrared spectrum of PSf, the stretching vibration characteristic absorption peak of aromatic (v C=C) bond appeared at 1625 cm^−1^ of PSf-OH spectrum, and the characteristic adsorption peak at 710–690 cm^−1^ was weaker. It can be seen that the substitution reaction on the benzene ring of the polysulfone membrane is generated at the benzene ring position 2. The results indicated that the DBPh grafting reaction occurred on the PSf membrane surface. For further grafting SDHPCS on the surface of polysulfone membrane, compared with the infrared spectrum of PSf, the surface spectrum of PSf-SDHPCS membranes showed new bond-stretching vibration characteristic absorption peaks at 979 cm^−1^, 934 cm^−1^, 913 cm^−1^, 1446 cm^−1^, and 3362 cm^−1^. Among them, the stretching vibration characteristic absorption peak of -OH was found at 3362 cm^−1^, which suggested that it might be -OH on DBPh that had not been completely grafted. Among them, 979 cm^−1^, 934 cm^−1^, and 913 cm^−1^ are the characteristic absorption peaks of the ether bond (C-O) stretching vibration, while the characteristic peaks of S=O stretching vibration in SDHPCS appear at 1446 cm^−1^. Therefore, it can be inferred that the DBPh functional group was successfully grafted on the surface of polysulfone membrane by UV grafting. Then, the anticoagulant molecule SDHPCS was successfully grafted onto the membrane surface by chemical reaction.

#### 3.1.4. XPS Analysis

XPS was used to confirm the components of the PSf, PSf-OH, and PSf-SDHPCS to further affirm the process of surface modification. As shown in Table 1, PSf-OH_60_ and PSf-OH_90_ is compared with PSf, the content of O slightly decreases (22.70% to 20.62%), and the content of C slightly increases (74.11% to 76.46%), mainly due to the DBPh that is grafted on the surface of the PSf membrane. In Figure 3b, XPS is used to detect the change in the element content in the modification process and the chemical composition and element composition of the surface PSf, PSf-OH, and PSf-SDHPCS membranes. In the PSf membrane, the main peaks of C1s, O1s, S2s, and S2p appear at PSf of 284.80 eV, 532.17 eV, 231.19 eV, and 168.30 eV, which are consistent with the elements contained in the polysulfone membrane material. After the UV grafting of the DBPh functional group, the XPS spectrum of PSf-OH is almost consistent with the PSf spectrum, mainly because the grafted DBPh only contains the three elements of C, O, and H, there is no new element content. Then, after the introduction of SDHPCS into PSf-OH, the main peak of N1s appeared in 400.00 eV, which mainly came from the -NH- group at C_6_ on the SDHPCS molecular chain, and at the same time, there was -SO_3_H on the SDHPCS chain, which also provided the element S. According to the element content, after the grafting of SDHPCS, the content of the S element in the modified membrane also increased significantly, indicating that SDHPCS was successfully grafted onto the surface of the polysulfone membrane.

Figure 3c–e shows the C1s peak curve fitting of PSf, PSf-OH, and PSf-SDHPCS. The results showed that when the PSf membrane is irradiated by ultraviolet light (254 nm/365 nm), BPh will undergo π-π * transition and be excited to reach singlet state S. It then rapidly transitions to a trilinear state, snatching hydrogen atoms from the adjacent polysulfone membrane surface and covalently linking to the membrane surface. A new O-C=O binding energy appears in C1s of PSf-SDHPCS, indicating that SDHPCS was grafted to the membrane surface. Figure 3f–h shows the S2p peak curve fitting of PSf, PSf-OH, and PSf-SDHPCS. The S2p peak curve fitting of PSf and PSf-OH is almost the same, mainly due to the introduction of a new S-binding bond. However, when SDHPCS was grafted to the surface of PSf membrane, -SO_3_H group was introduced. Although all of them were S=O bonds, the intervention of SDHPCS affected the binding energy of S=O bonds of the modified membrane.

#### 3.1.5. The Across-Section Morphologies of Membranes

**Figure 4:**
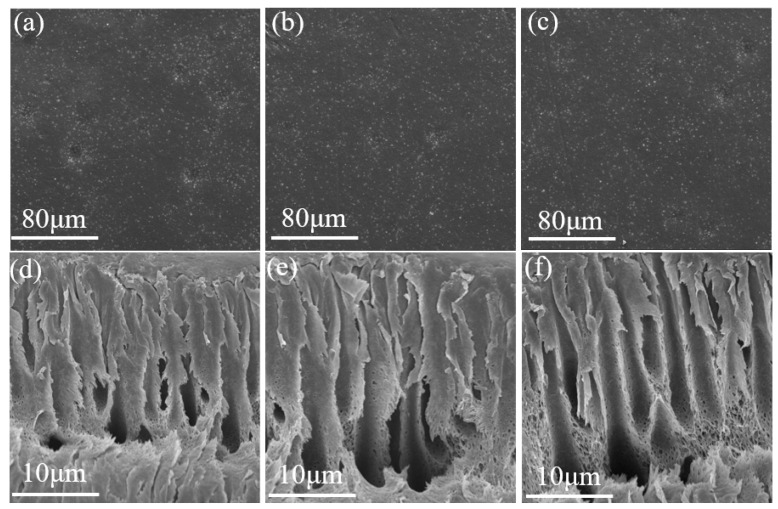
The SEM images (**a**–**c**) PSf, PSf-OH, and PSf-SDHPCS membranes surface; the SEM image of cross section (**d**–**f**) of PSf, PSf-OH, and PSf-SDHPCS membranes.

The SEM images of the across-sections of PSf, PSf-OH, and PSf-SDHPCS membranes are shown in Figure 4. The polysulfone membrane itself has good mechanical properties, high temperature properties, as well as its unique porous intermediate layer and dense adjustable bottom layer composition. The removal of the hemodialysis membrane needs to improve the blood compatibility of the membrane surface, which is very important for purifying the harmful low and medium molecules in the blood and waste generated by metabolism, that is, the permeability of the polysulfone membrane body. Therefore, on the premise of the modification of the membrane surface, the cellular structure of the pore size of the polysulfone membrane itself is not cracked. For this reason, the cross section morphology of the modified polysulfone membrane was analyzed by SEM. The cross sections of all the membranes showed an asymmetric porous finger-like structure, which was mainly composed of a dense layer on the upper part and a loose layer on the lower part. Therefore, the modified membrane does not change the cross-section structure of the PSf membranes.

### 3.2. Hemocompatibility Tests

#### 3.2.1. Adsorption of Protein

It has been reported that the contact of the foreign body surface with blood mainly results in the activation of the coagulation pathway in vivo [26]. According to the waterfall sequence coagulation mechanism proposed by Davies, the adsorption of blood protein is the first step of coagulation. After platelet adhesion, various coagulation pathways are further activated, and finally lead to the occurrence of thrombosis genes [27]. In Figure 2b, the protein adsorption capacity on the surface of the modified polysulfone membrane was significantly lower than that of the unmodified polysulfone membrane. The adsorption capacity of the UV-grafted PSf-OH membrane decreased from 328 μg·cm^−2^ to 244 μg·cm^−2^, and the decrease in the protein adsorption capacity on the surface of the membrane was mainly due to DBPh grafted on the surface of the polysulfone membrane. The hydrophilicity of the membrane surface was changed by introducing the -OH group on the membrane surface, which was conducive to improving the protein adsorption on the membrane surface. The anchorage active group provided by DBPh was grafted with SDHPCS, and the membrane surface was provided with a long chain molecular brush and the -SO_3_H group was provided at the end. The protein adsorption capacity on the membrane surface was reduced to 32 μg·cm^−2^, mainly due to the electrostatic repulsion between the negatively charged -SO_3_H group and the negatively charged BSA protein. The contact between the protein and the membrane surface is prevented, thus reducing the adsorption of the BSA protein on the surface of the PSf-SDHPCS membrane.

#### 3.2.2. Platelet Adhesion

**Figure 5:**
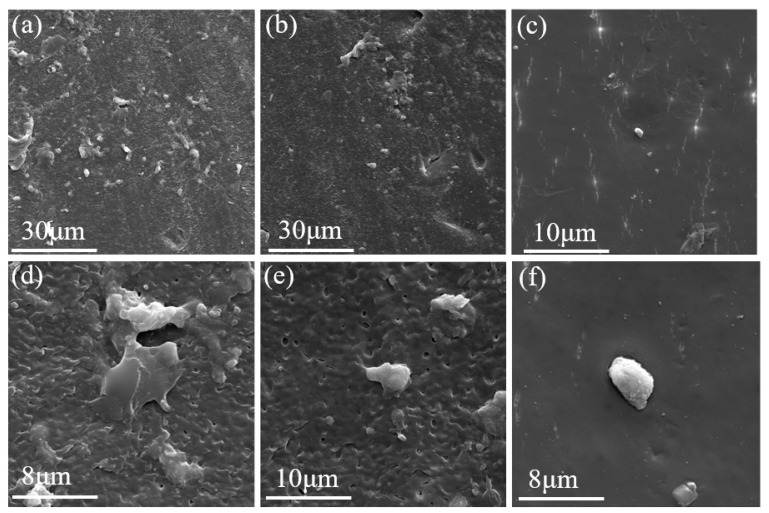
The SEM images (**a**–**f**) of PSf, PSf-OH, and PSf-SDHPCS membranes with adsorbed platelets. Magnification: 1000× (**a**–**c**); magnification: 5000× (**d**–**f**).

After activation, platelets will deform from round platelets to flat platelets, then to irregular platelets, and then protruding “false feet”. The entire deformation process is accompanied by platelet aggregation. After the platelets deform and aggregate on the surface of the material, they can activate various coagulation factors, leading to thrombosis [28,29]. When platelets undergo morphological changes and aggregate on the surface of the material, various clotting factors will be activated, thus promoting the formation of thrombi. To this end, we studied the number and morphology of platelet adhesions on PSf, PSf-OH, and PSf-SDHPCS membrane surfaces. The results are shown in Figure 5. The results showed that the number of platelets adhering to the PSf-SDHPCS membrane was much less than that on the PSf and PSF-OH membranes. On the surface of the PSf and PSf-OH membranes, platelets were observed to appear as flat or irregular in shape and to extend “pseudopods” to form thrombi. However, no platelet deformation and “pseudopodia” were found on the surface of the PSf-SHPCS membrane, indicating that the PSf-SHPCS membrane effectively inhibited platelet adhesion and deformation. This indirectly indicates that the PSf-SHPCS membrane has good blood compatibility.

#### 3.2.3. Hemolysis Assay

**Figure 6:**
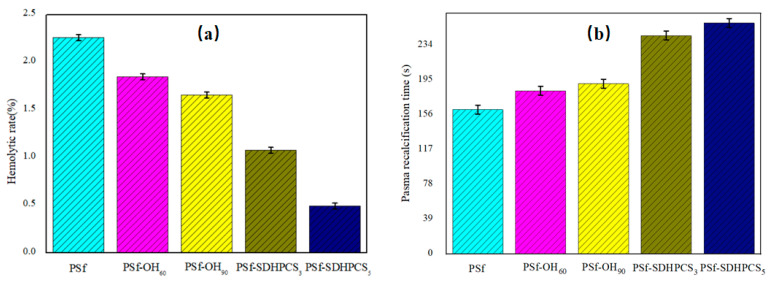
The hemolysis ratios (**a**) and plasma recalcification times (**b**) of PSf, PSf-OH, and PSf-SDHPCS membranes.

Hemolysis (HR) is defined as the release of hemoglobin and adenosine diphosphate into the plasma due to damage to the erythrocyte membrane. TADP causes platelet activation, leading to thrombosis. Therefore, the hemolysis rate is an important index to evaluate both the blood compatibility of membrane materials, and the degree of breakage of red blood cells in blood. The test result has the advantages of high accuracy, direct measurement, simplicity, and speed, and has become the only index to evaluate the blood compatibility of materials in national standards and norms. It is also one of the main indexes to evaluate whether materials can be used in the biomedical field [26,30]. At present, the hemolysis rate of the membrane material is less than 5%, which is in line with the requirements for the hemolysis rate of medical materials, otherwise the material will cause hemolysis and other risks. Figure 6a shows the changes in the hemolysis rate on the membrane surface of PSf, PSf-OH, and PSf-SDHPCS membranes. The results showed that the HR of PSf membrane and modified polysulfone membrane both meet the requirements of ISO 10993 [31] for the HR of blood compatibility materials in vitro experiments to be less than 5%. In turn, the graft of DBPh and SDHPCS on the PSf membrane surface improved the anti-hemolysis performance of the membrane surface. After the graft of SDHPCS, the hemolysis rate on the PSf membrane surface was greatly reduced, mainly because the graft of SDHPCS improved the hydrophilicity of the membrane surface and formed a hydration layer between the interface of red blood cells and the membrane surface, which effectively protected red blood cells. The membrane surface damage to red blood cells is reduced.

#### 3.2.4. Plasma Recalcification Time (PRT)

The blood recalcification time test is a method to simulate the mechanism of endogenous coagulation process in vitro, which is relatively simple and direct for evaluating the effect of membrane materials on endogenous coagulation. The test results of the blood recalcium time are shown in Figure 6b. The results showed that after DBPh grafting on the surface of the membrane, the blood calcium recombination time on the surface of the modified polysulfone membrane was not extended significantly compared with that on the surface of the unmodified polysulfone membrane. After SDHPCS grafting, the blood calcium recombination time on the surface of the modified polysulfone membrane was extended from the original 162 s to 259 s, and the grafting density on the surface of the membrane increased with the extension of grafting time. The time of blood recalcification was also prolonged, mainly because after grafting SDHPCS, a large number of -SO_3_H groups were introduced on the membrane surface, which could bind with antithrombin, thrombin factor Xa, and other factors. After binding with antithrombin, the production of thrombin antithrombin (TAT) and Xa antithrombin complex was accelerated, resulting in the inactivation of clotting factor. This process stops the clotting from happening.

#### 3.2.5. APTT, PT and TT

**Figure 7:**
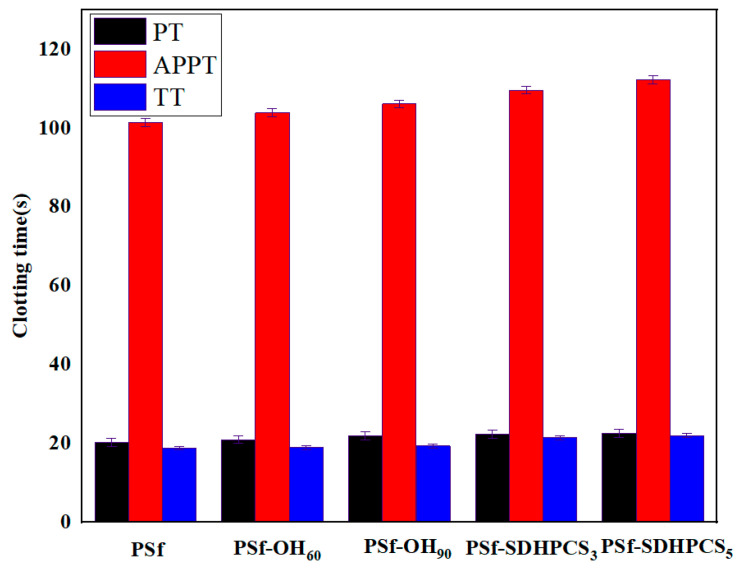
APTT, PT, and TT of PSf, PSf-OH, and PSf-SDHPCS membranes.

APTT, PT, and TT tests can evaluate the anticoagulation ability of biomedical materials. Among them, APTT is used to evaluate the clotting pathway of endogenous factors, while PT is used to evaluate the clotting pathway of exogenous factors, and TT is an index for the determination of human plasma thrombin time in vitro. Figure 7 shows the APTT, PT, and TT of PSf, PSf-OH, and PSf-SDHPCS membranes. The results showed that the APTT, PT, and TT of the PSf-OH membrane were significantly improved (106.1, 21.9, and 19.4 s, respectively) compared to the PSf membrane (101.4, 20.3, and 18.8 s, respectively). This indicates that the hydrogen group has a certain anticoagulation effect after grafting to the membrane surface. In addition, all PSf-SDHPCS membranes showed greater APTT values when compared with PSf, which is due to the fact that SDHPCS grafts on the PSf membrane surface affect the endogenous coagulation pathway and have a certain degree of anticoagulation properties. At the same time, compared with the PSf membrane, we also found that the PT and TT values in PSf-SDHPCS_5_ membrane were increased.

## 4. Conclusions

The blood compatibility and antibacterial activity of synthetic dihydroxy propyl sulfonated chitosan and modified polysulfone were systematically evaluated based on the coagulation mechanism or the conditions leading to the discovery of the clotting phenomenon. The effects of protein adsorption, hemolysis, blood recalcium time, platelet adhesion, coagulation testing, and a synthetic membrane on the activity of Staphylococcus aureus were mainly carried out. Compared with the PSf membrane, the protein adsorption capacity of the PSf-SDHPCS membrane was reduced by 90.24%. The developed, modified membrane had good anti-protein adsorption properties, and the negatively charged -SO_3_H group was identified, which formed an electrostatic repulsion with the protein and prevented the adsorption of a large amount of fibrinogen on the surface of the modified membrane. Platelets are the core of thrombosis, and inhibition of platelet adhesion, aggregation, and activation may be the key to inhibiting the thrombosis of biomaterials. According to the studies, the amount of platelet adhesion on the modified polysulfone membrane surface decreased, which was related to the increased hydrophilicity of the modified membrane surface, which reduced the interface energy of the membrane surface and did not activate platelets. This prevents the adhesion and destruction of the platelets on the membrane surface. The HR of the PSf membrane and modified polysulfone membrane both meet the requirements of ISO 10993 [31] for an HR of less than 5% of in vitro blood compatible materials. After the graft of SDHPCS on the PSf membrane surface, the hemolytic property of the PSf membrane surface was improved, indicating that the modified polysulfone membrane had good stability of red blood cells. According to the total coagulation test, the APTT, PT, and TT of PSf-SDHPCS membrane showed significant improvement, which were 112.2 s, 22.6 s, and 22.0 s, respectively. An SDHPCS graft onto the surface of PSf membrane would affect the endogenous coagulation pathway, prolonging the activation of prothrombin and blocking thrombosis.

## Figures and Tables

**Table 1:** The elemental surface compositions of the membranes determined by XPS analysis of PSf, PSf-OH, and PSf-SDHPCS.

Samples	Elemental (at%)			
	O%	N%	C%	S%
PSf	22.70	0.18	74.11	2.62
PSf-OH_60_	21.93	0.14	75.72	2.20
PSf-OH_90_	20.62	0.21	76.46	2.66
PSf-SDHPCS_3_	30.24	2.88	62.99	3.94
PSf-SDHPCS_5_	33.41	3.31	59.17	4.11

## Data Availability

Data are contained within the article.
